# Age-Related Clinicopathologic Patterns in Ewing Sarcoma (FET::ETS Family): A Comparative Analysis of Pediatric and Adult Patients

**DOI:** 10.3390/cancers18010133

**Published:** 2025-12-30

**Authors:** Rola H. Ali, Eiman M. A. Mohammed, Amir A. Ahmed, Ahmad R. Alsaber, Hind S. Al-Otaibi, Samer A. K. Abdulmoneim, Abdulaziz Hassan, Fatemah Almousawi, Nisreen Khalifa, Abdullah A. Ali, Shakir Bahzad, Fahad G. Alenezi, Muath AlNassar, Abdulaziz AlJassim

**Affiliations:** 1 Department of Pathology, College of Medicine, Kuwait University, Safat 13110, Kuwait; fatemah.almousawi@ku.edu.kw; 2 Histopathology Laboratory, Sabah Hospital, Sabah Medical District, Safat 13001, Kuwait; 3 Molecular Genetics Laboratory, Kuwait Cancer Center, Sabah Medical District, Safat 13001, Kuwait; emohammad@moh.gov.kw (E.M.A.M.); aaahmed@moh.gov.kw (A.A.A.); sbahzad@moh.gov.kw (S.B.); 4 Department of Management, College of Business and Economics, American University of Kuwait, Safat 13034, Kuwait; aalsaber@auk.edu.kw; 5 Department of Surgical Oncology, Kuwait Cancer Center, Sabah Medical District, Safat 13001, Kuwait; hs.alotaibi@moh.gov.kw (H.S.A.-O.); abdulmoneim0933@moh.gov.kw (S.A.K.A.); 6 Department of Diagnostic Radiology, Jaber Alahmad Hospital, Safat 13001, Kuwait; abdulazizhassan1989@gmail.com; 7 Department of Pediatric Oncology, NBK Children’s Hospital, Sabah Medical District, Safat 13001, Kuwait; khalifa.nisreen@gmail.com (N.K.); aa.ali@moh.gov.kw (A.A.A.); 8 Department of Medical Oncology, Kuwait Cancer Center, Sabah Medical District, Safat 13001, Kuwait; falenezi05@gmail.com (F.G.A.); muathalnassar@gmail.com (M.A.); azaljassim@moh.gov.kw (A.A.)

**Keywords:** Ewing sarcoma, FET-ETS, Ewing-like, pediatric oncology, old adults, extraskeletal, RNA sequencing

## Abstract

**Background:** Ewing sarcoma (ES) is a rare, aggressive small round cell sarcoma (SRCS) that peaks in adolescence. Given its rarity, atypical age or site presentations increase the risk of misclassification. This study examines age-related clinicopathological patterns in molecularly confirmed canonical ES (FET::ETS-fused). **Methods:** Between 2016 and 2025, 90 tumors diagnosed as ES or Ewing-like SRCSs underwent targeted RNA sequencing and/or *EWSR1* break-apart fluorescence in situ hybridization. Patients were stratified into three age groups: 0–18, 19–39, and ≥40 years. Clinical, anatomical, pathological, molecular, and treatment/outcome variables were compared across strata. **Results:** Canonical ES accounted for 84% (76/90) of SRCSs, dominated by *EWSR1::FLI1* (89%). ES comprised 91% of SRCSs in children but declined to 75% in older adults. Tumors arose mainly in bone (63%), with a significant age association (*p* = 0.016): children and young adults were primarily skeletal (73% and 62%), whereas older adults were predominantly extraskeletal (78%). Renal ES clustered in adults ≥40 years (*p* = 0.003). Classic histology predominated; atypical patterns were more common in extraskeletal tumors but lacked age specificity. Ewing-like SRCSs (*n* = 14), with heterogeneous or absent fusions, displayed a broader age distribution—including infants and older adults—and a marked extraskeletal predominance (86%, *p* = 0.001). Metastatic presentation strongly predicted inferior survival (*p* = 0.025). Treatment was multimodal, with neoadjuvant chemotherapy more frequent in children (90%, *p* = 0.029). **Conclusions:** Age significantly influences anatomic presentation and certain treatment choices in ES, whereas histology and survival remain broadly similar across groups. Age-linked extraskeletal trends reinforce the importance of routine molecular testing, particularly in underreported Middle Eastern populations.

## Simple Summary

Ewing sarcoma is a rare and aggressive cancer of bone and soft tissue that most often affects teenagers. Because it is uncommon and can arise in many different parts of the body, it can be difficult to recognize—particularly when it occurs outside the typical age range or in unusual anatomical sites. This challenge is especially relevant in regions where molecular diagnostic testing is relatively new or inconsistently available, which can complicate accurate diagnosis. In this study, we compared clinical, anatomical, pathological, molecular, treatment, and outcome features of Ewing sarcoma across three age groups: children (0–18 years), adolescents/young adults (19–39 years), and older adults (≥40 years). We identified clear age-related patterns, including a shift from predominantly bone tumors in younger patients to predominantly soft-tissue tumors in older adults. These insights can improve diagnostic accuracy, guide clinical decision-making, and strengthen regional understanding of this rare malignancy.

## 1. Introduction

Ewing sarcoma (ES) is an uncommon, aggressive bone and soft-tissue small round cell sarcoma (SRCS) that predominantly affects children and adolescents. Incidence peaks during the second decade of life, with approximately 80% of cases diagnosed before age 20 and 20–30% occurring in the first decade of life. Incidence declines sharply thereafter and is exceedingly rare in older adults [1,2]. Age at diagnosis has long been recognized as an important prognostic factor, with older patients more frequently exhibiting adverse clinical features and inferior survival outcomes [3,4].

Advances in molecular genetics have refined the definition of ES, which is now strictly characterized by FET::ETS fusions—most commonly *EWSR1::FLI1*—thereby distinguishing it from the heterogeneous group of “Ewing-like” sarcomas that lack these canonical rearrangements [5,6]. This latter category includes tumors with *EWSR1*–non-ETS fusions [7], *CIC* rearrangements [8,9], and *BCOR* alterations [10], each representing a biologically distinct entity with characteristic age distributions, anatomic predilections, and prognostic profiles [11]. This molecular reclassification is particularly important when evaluating age-related differences, as older series predating routine fusion testing likely incorporated a substantial proportion of Ewing-like mimics, thereby complicating interpretation of historical epidemiologic and survival data [12].

Given these refinements in disease classification, the present study aimed to delineate age-associated clinicopathological features in a national, fully molecularly validated SRCS cohort (*n* = 90) treated at Kuwait’s two tertiary cancer centers over the past decade. The cohort includes 76 canonical ES cases and a smaller comparative subset of 14 Ewing-like SRCSs, allowing limited side-by-side evaluation where appropriate. This provides a contemporary dataset from an underrepresented region where routine molecular diagnostics have only recently gained momentum. By restricting the primary analysis to genetically confirmed ES, the study offers a clearer depiction of true age-linked patterns while minimizing the diagnostic noise that historically confounded older series.

## 2. Materials and Methods

### 2.1. Patient Selection

We retrospectively reviewed all undifferentiated SRCSs that underwent molecular testing at the Molecular Genetics Laboratory, Kuwait Cancer Center, between 2016 and 2025. The search encompassed canonical ESs (FET::ETS-rearranged) as well as Ewing-like mimics with alternative gene rearrangements. Cases lacking molecular studies, those with failed results, or those with insufficient material were excluded. Both adult cases from Kuwait Cancer Center and pediatric referrals from NBK Children’s Hospital were included.

Clinical and pathological data—including age, gender, anatomic site, tumor size, stage, treatment details, and follow-up outcomes—were retrieved from electronic medical records. Patients were categorized into three age groups for analysis: children (≤18 years), adolescents and young adults (19–39 years), and older adults (≥40 years). Hematoxylin and eosin (H&E) slides were reviewed by a soft-tissue pathologist (RHA) for case assessment and inclusion, with evaluation of architectural patterns, cytologic features, stromal characteristics, necrosis, and mitotic activity. Immunohistochemical markers were evaluated whenever available, including CD99 and NKX2.2, along with additional markers used in the differential diagnosis of small round cell neoplasms (e.g., epithelial, neural, myogenic, and melanocytic markers).

A radiologist reviewed pre-operative imaging to determine the tumor site of origin, particularly in equivocal cases. Tumors were designated “skeletal” when centered within the bone, with or without soft-tissue extension. Tumors arising in soft tissue or visceral organs were classified as extraskeletal, even if secondarily invading bone. Skeletal tumors were subclassified as axial (spine, pelvis, sacrum, chest wall/rib, scapula/shoulder girdle) or appendicular (long and short bones of the extremities), following established ES conventions. Selected figures illustrating representative imaging and histologic features are included for contextual purposes only and did not form the basis of any statistical analyses.

### 2.2. Fluorescence In Situ Hybridization (FISH)

FISH analysis for *EWSR1* (22q12) and *FUS* (16p11) rearrangements was performed on 87 formalin-fixed, paraffin-embedded (FFPE) samples using dual-color break-apart probes (Abbott Molecular/Vysis, Abbott Park, IL, USA). Four-micrometer sections were deparaffinized and processed using standard pretreatment protocols, including heat-induced retrieval, protease digestion, and ethanol dehydration. Approximately 20 µL of probe was applied, followed by co-denaturation at 85 °C for 5 min and overnight hybridization at 37 °C (ThermoBrite, Abbott Molecular/Vysis, Abbott Park, IL, USA).

Post-hybridization washes were performed in 2× SSC/0.3% NP-40 at 72 °C, and slides were counterstained with DAPI. Tumor-rich areas were identified by correlation with H&E sections. A minimum of 100 non-overlapping nuclei were evaluated using a Zeiss fluorescence microscope. Normal nuclei demonstrated two fused (yellow) signals; rearranged nuclei typically showed one fused and one separated orange–green pair. A split signal was defined as probe separation greater than two signal diameters. A result was considered positive when ≥20% of nuclei demonstrated split signals.

### 2.3. Targeted Next Generation Sequencing

Targeted RNA-based fusion analysis was performed on FFPE tissue from 71 patients. RNA extraction was performed using the RecoverAll Total Nucleic Acid Isolation Kit (Thermo Fisher Scientific, Waltham, MA, USA). Concentration and integrity were assessed using a Qubit 3.0 Fluorometer (Thermo Fisher Scientific, Waltham, MA, USA), Agilent TapeStation (Agilent Technologies, Santa Clara, CA, USA), and the Archer PreSeq RNA QC assay (Integrated DNA Technologies, Inc., Coralville, IA, USA); samples with Ct > 28 were excluded. Ideally, 200 ng of RNA was used for library preparation, although samples with ≥50 ng were also included.

Libraries were prepared using the Archer FusionPlex Sarcoma Panel (v2) according to manufacturer protocols. The Anchored Multiplex PCR (AMP™, ArcherDX, Boulder, CO, USA), method employs unidirectional gene-specific primers to detect both known and previously unreported fusions [13]. The panel targets 62 sarcoma-associated genes with 659 primer pairs. Sequencing was performed on the Ion Torrent S5 XL platform (Thermo Fisher Scientific).

Data were analyzed using Archer Analysis v5.0.4. High-confidence fusion calls required ≥5 unique breakpoint-spanning reads and ≥3 reads with distinct start sites. Assay sensitivity was optimized for canonical oncogenic isoforms but reduced in samples with <10% tumor cellularity or degraded RNA. All runs were evaluated for key quality-control parameters, including mapped reads and duplication rates.

FISH and targeted RNA-NGS were used in a complementary manner based on tissue availability and diagnostic indication; molecular assay type was not used as a variable in statistical analyses.

### 2.4. Statistical Analysis

Descriptive statistics summarized frequency distributions, central tendency (mean, median) and variability (range, standard deviation). Graphical displays were used where appropriate. Comparisons were performed using univariate analyses; categorical variables were evaluated using Pearson’s chi-squared test and continuous variables using independent two-sample *t*-tests. Survival was assessed using the Kaplan–Meier method. Overall survival (OS) was defined from date of diagnosis to death or last follow-up; progression-free survival (PFS) from diagnosis to first radiologic/clinical metastasis or local recurrence, with patients censored at last follow-up if no event occurred. All analyses were conducted using JAMOVI (v2.5.7.0), with two-tailed *p*-values < 0.05 considered statistically significant.

## 3. Results

### 3.1. Cohort Characteristics and Age Subgroup Analyses

**Figure 1:**
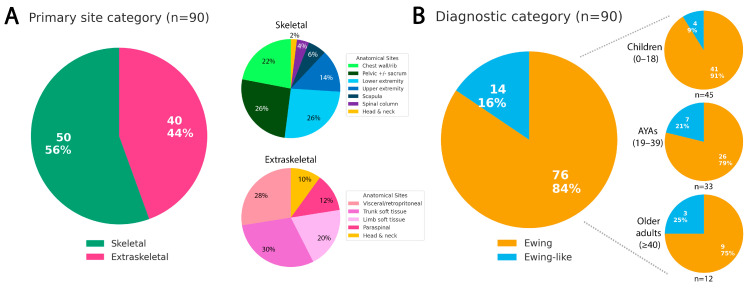
Primary site and diagnostic category distributions in 90 small round cell sarcomas. (**A**) Primary site distribution with detailed anatomical breakdowns of skeletal (*n* = 50) and extraskeletal (*n* = 40) tumors. (**B**) Distribution of canonical Ewing sarcoma (FET::ETS-rearranged) and Ewing-like sarcomas, displayed overall and across age groups.

From 2016 to 2025, we identified 45 pediatric patients ≤ 18 years and 45 adult patients > 18 years diagnosed with ES and Ewing-like SRCSs that underwent molecular testing at our institution. The cohort comprised 43 females and 47 males (F:M = 0.92:1). Fifty tumors originated in skeletal sites and 40 in extraskeletal locations (Figure 1A). Clinicopathological characteristics are summarized in Table 1.

A diagnosis of canonical Ewing family (FET::ETS-rearranged) sarcoma was confirmed in 84% (76/90) of cases based on RNA sequencing and/or FISH results interpreted within the appropriate histopathological context. Among these, 58% (44/76) were fusion-positive by sequencing, while 42% (32/76) demonstrated *EWSR1* rearrangement by FISH alone. An overview of molecular testing modalities across the cohort is provided in Supplementary Table S1. Although break-apart FISH does not identify a fusion partner, these cases were supported by classic ES morphology and immunophenotype. Concordance between sequencing and FISH was 85% (40/47), with seven discordant cases (15%) attributed to technical limitations such as scant or necrotic tissue. Among fusion-positive ES tumors, *EWSR1::FLI1* was predominant (39/44; 89%), followed by *EWSR1::ERG* (4/44; 9%) and *FUS::ERG* (1/44; 2%).

Within the ES group (*n* = 76), there were 37 females and 39 males (F:M = 0.95:1). This included 41 children ≤ 18 years (median 13.4 years) and 35 adults > 18 years (median 28.0 years), further stratified into 26 AYAs aged 19–39 years (median 25.3 years) and 9 older adults ≥ 40 years (median 46.3 years). A progressive decline in the proportion of ES among all SRCSs was observed with increasing age—91% in children vs. 75% in older adults—although this trend did not reach statistical significance (Figure 1B).

**Figure 2:**
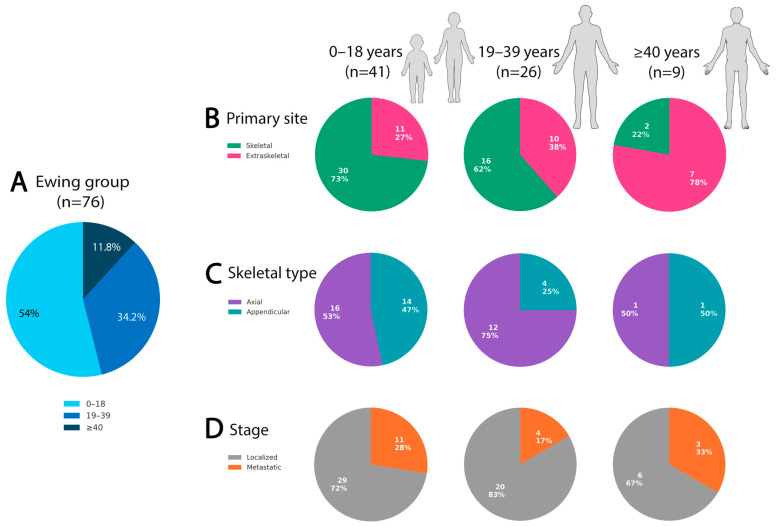
Clinicopathological characteristics of the Ewing sarcoma cohort stratified by three age groups (≤18, 19–39, and ≥40 years). (**A**) Age distribution of the cohort. (**B**) Distribution of primary tumor site (skeletal vs. extraskeletal) across age groups, demonstrating a relative increase in extraskeletal tumors with advancing age. (**C**) Skeletal tumor distribution (axial vs. appendicular) among osseous cases, showing a higher proportion of axial involvement in the 19–39 age group compared with children. (**D**) Stage at diagnosis (localized vs. metastatic) across age groups, without significant differences.

Most ESs originated in bone (63.2%, 48/76) rather than extraskeletal sites (36.8%, 28/76), a significant difference (*p* = 0.001). Primary site distribution also varied with age: skeletal tumors predominated in children (73.2%, 30/41) and AYAs (61.5%, 16/26) but were uncommon in older adults (22.2%, 2/9) (Table 2). Conversely, extraskeletal disease increased steadily with age—26.8% (11/41), 38.5% (10/26), and 77.8% (7/9), respectively (Figure 2).

**Figure 3:**
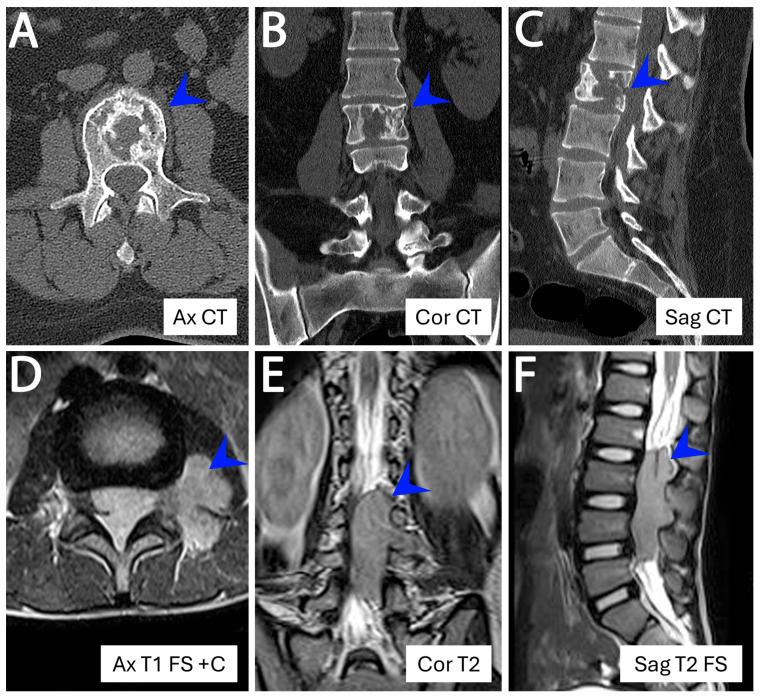
Contrasting two *EWSR1::FLI1*–positive Ewing sarcomas of the spinal region. (**A**–**C**) Skeletal tumor arising from the L2 vertebral body in a 24-year-old man. (**D**–**F**) Extraskeletal extradural tumor in the paraspinal soft tissue with foraminal extension and infiltration of surrounding muscles, in an 8-year-old boy. Ax = axial; Cor = coronal; Sag = sagittal; CT = computed tomography; T1 = T1-weighted MRI; T2 = T2-weighted MRI; FS = fat saturation; C = contrast.

**Figure 4:**
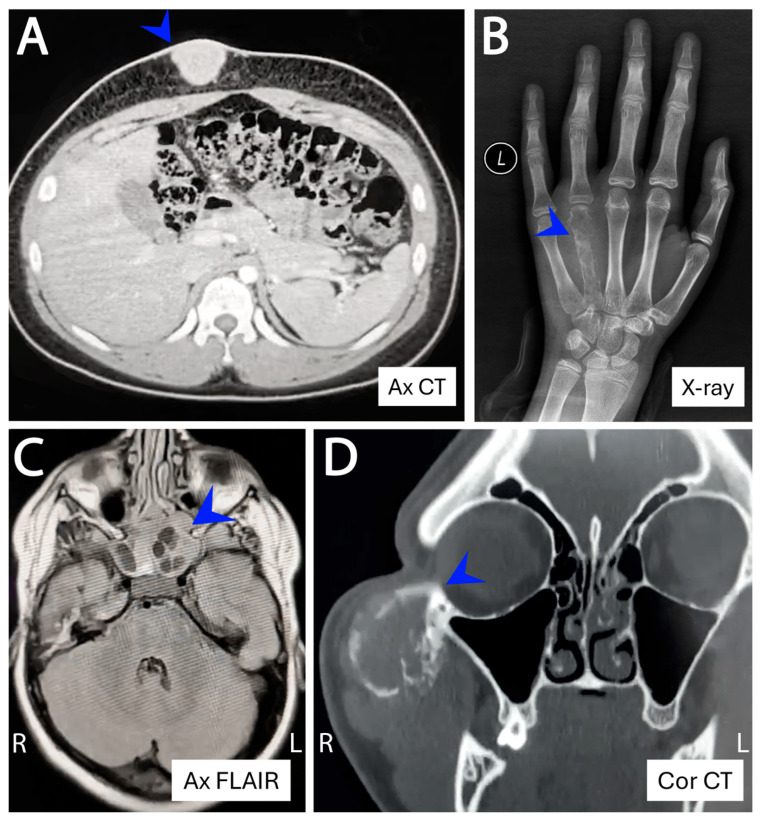
Ewing sarcomas at unusual locations. (**A**) Subcutaneous lesion at the epigastric region in a 14-year-old male. (**B**) Skeletal tumor of the 4th metacarpal bone in a 12-year-old female. (**C**) Head and neck lesion at the sphenoid–ethmoid sinuses and nasopharyngeal space in a 9-year-old boy. (**D**) Lesion arising from the zygomatic arch in a 25-year-old man. Ax = axial; Cor = coronal; CT = computed tomography; FLAIR = fluid-attenuated inversion recovery MRI.

**Figure 5:**
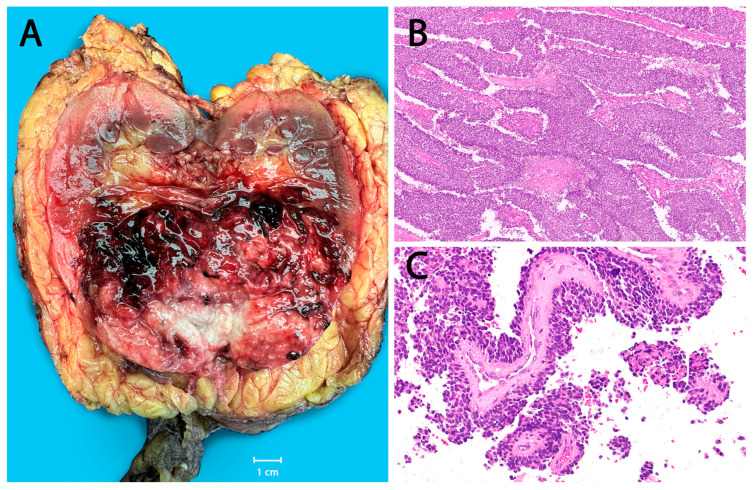
Renal Ewing sarcoma. (**A**) Hemorrhagic necrotic tumor in the lower pole of the kidney, harboring *FUS::ERG* fusion, in a 30-year-old male. (**B**) Histology of an *EWSR1::FLI1* fused tumor in a 40-year-old male, with classic histology and geographic necrosis (scanning mag.). (**C**) Another *EWSR1::FLI1* fused tumor in a 52-year-old male with unusual pseudo-papillomatous pattern mimicking carcinoma (20×).

Restricting analysis to skeletal ESs (*n* = 48), 60.4% (29/48) arose in axial locations and 39.6% (19/48) in appendicular bones. Axial involvement was most common in AYAs (12/16, 75.0%) compared with children (16/30, 53.3%) and older adults (1/2, 50%), though these differences were not statistically significant. The most frequent skeletal sites—pelvis, chest wall/rib, spine, femur, and tibia—did not differ significantly across age groups (Figure 3A–C). In contrast, extraskeletal locations were widely distributed in somatic soft tissues and visceral sites, where imaging findings became non-specific out of the bone context and could not predict the diagnosis in the majority of cases (Figure 3D–F). At such rare extraskeletal or skeletal sites (e.g., superficial soft tissue, visceral locations, acral), the tumor often posed diagnostic challenges, radiologically and pathologically, and frequently required extensive immunohistochemical workup and molecular confirmation and also required prior experience and knowledge to suspect ES at these sites (Figure 4). Renal ES, a rare but recognized extraskeletal presentation, showed a notable age predilection with 3 of 5 renal ESs occurring in adults ≥ 40 years (*p* = 0.003) (Figure 5).

**Figure 6:**
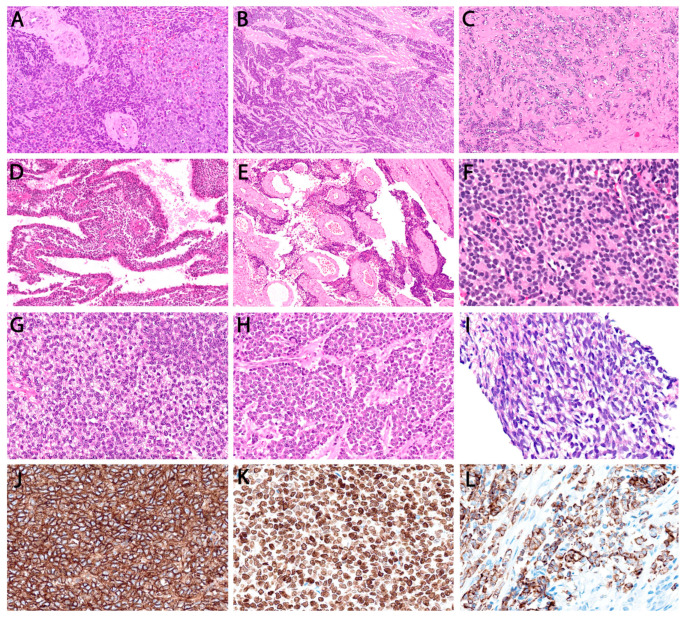
Cytoarchitectural patterns in canonical Ewing family (FET::ETS-rearranged) sarcoma. (**A**) Classic sheet-like architecture with light- and dark-cell appearance. (**B**) Unusual nested arrangement with fibrous septa. (**C**) Unusually accentuated fibrous matrix. (**D**) Rare multicystic pattern with epithelioid lining mimicking biphasic synovial sarcoma. (**E**) Perivascular/peudopapillary pattern. (**F**) Classic uniform round cells arranged in pseudo-rosettes. (**G**) Clear cell change. (**H**) Atypical Ewing with epithelioid cells and small prominent nucleoli. (**I**) Unusual rare spindling. (**J**) Characteristic strong and diffuse membranous CD99 staining. (**K**) Expected diffuse NKX2.2 nuclear staining. (**L**) Focal to patchy expression of cytokeratin AE1/3—a pitfall.

Histologic parameters—including growth pattern and nuclear cytomorphology—did not demonstrate significant age-related variation, indicating that the microscopic appearance of canonical ES is largely conserved across pediatric, AYA, and older adult groups. Classic architectural arrangement with solid sheets of uniform small round cells predominated with pseudo-rosette formation identified in 27.6% (21/76). Figure 6 provides morphologic examples encountered in this cohort for contextual purposes. Rare architectural patterns were observed, particularly in extraskeletal tumors, including nesting/trabecular growth with collagenous septa (21%, 16/76), perivascular/pseudopapillary formations (7.9%, 6/76), and multicystic change (2.6%, 2/76). Typical uniform nuclear morphology was similarly consistent across age groups; atypia (enlarged nuclei, irregular contour, conspicuous nucleoli) was reported in 17.1%, 19.2%, and 33.3% of the respective age categories. Immunohistochemically, diffuse CD99 and NKX2.2 expression remained characteristic (*p* < 0.001). Focal or patchy positivity for cytokeratin (25%) and S100 (21%) represented recognized diagnostic pitfalls.

**Figure 7:**
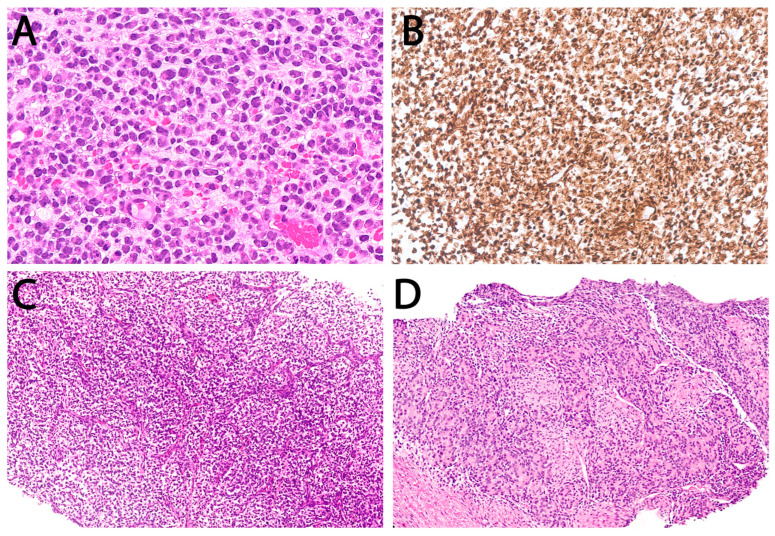
“Ewing-like” sarcomas. (**A**) *CIC::DUX4* sarcoma, undifferentiated round cells with nuclear pleomorphism (40×). (**B**) Characteristic WT1 nuclear staining in *CIC::DUX4* sarcoma. (**C**) Infantile BCOR-altered sarcoma, ovoid to short spindle cells in a myxoid stroma and rich capillary network (10×). (**D**) *YWHAE::NUTM2B* sarcoma, another infantile case, mixture of round cells with rosette formation and short spindle cells (10×).

“Ewing-like” sarcomas were identified in 14/90 SRCSs. Nine cases demonstrated distinct non–FET::ETS molecular alterations: *CIC::DUX4* (*n* = 5), *BCOR* alteration (*n* = 1), *EWSR1::ATF1* (*n* = 1), *EWSR1::CREB1* (*n* = 1), and *YWHAE::NUTM2B* (*n* = 1). The remaining five were fusion-negative and classified as undifferentiated SRCS-NOS. The Ewing-like group had a median age of 22 years (range 0.03–70) and an F:M ratio of 0.75:1. Most patients were adults >18 years (71.4%, 10/14), with a distribution centered around young adulthood rather than the adolescent peak typical of ES; only four (28.6%) were ≤18 years, though the age difference between ES and Ewing-like tumors was not statistically significant. A striking predominance of extraskeletal disease was observed (85.7%, 12/14; *p* = 0.001), which was markedly higher than that in canonical ES. Sites included trunk soft tissue (*n* = 4), extremities (*n* = 4), visceral/retroperitoneum (*n* = 3), and head and neck (*n* = 1). Only two tumors arose in bone (Figure 7).

### 3.2. Treatment and Outcome

Analysis focused on the canonical ES subgroup, which was relatively molecularly homogeneous and treated with standardized protocols. Most ES patients presented with localized disease (75%, 55/73), whereas one quarter were metastatic at diagnosis (25%, 18/73), with the lungs being the most common site of spread. Stage distribution did not differ significantly across the three age tiers (27.5%, 16.7%, and 33.3%, respectively) (Figure 2D) or across the binary age grouping (≤18 vs. >18). Ewing-like sarcomas demonstrated a numerically higher frequency of metastatic presentation compared with ES (46.2% vs. 24.7%); however, this difference did not reach statistical significance. Notably, the Ewing-like group showed significantly more frequent lymph-node involvement (50%; *p* = 0.002) than ES at any point in time.

**Figure 8:**
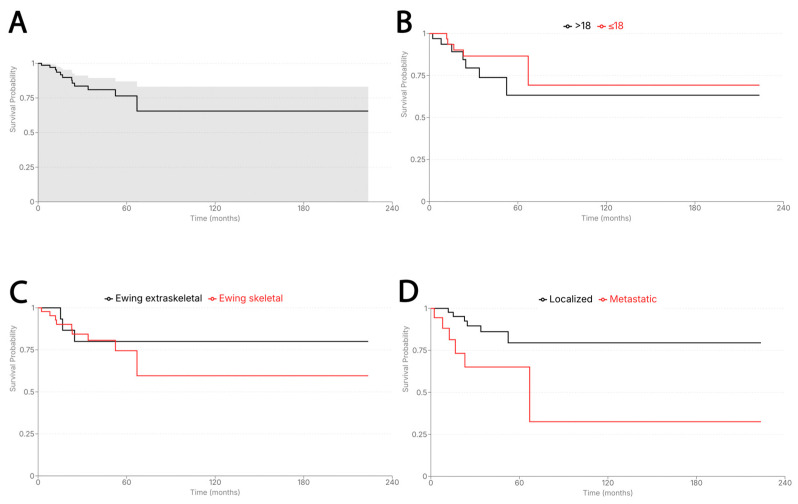
Kaplan–Meier curves for Ewing sarcoma. (**A**) Overall survival. (**B**) Age group comparison (≤18 vs. >18 years) showing no significant survival difference (*p* = 0.250). (**C**) Skeletal vs. extraskeletal primary sites demonstrating comparable outcomes (*p* = 0.625). (**D**) Metastatic presentation with significantly worse OS compared with localized disease (*p* = 0.025).

Median follow-up was 32.4 months (range 2.1–223.7 months). At last contact, 17% (12/71) of ES patients had died. Median OS for the cohort was not reached, with an estimated 60-month OS of approximately 76.5%, reflecting substantial censoring at later time points. Metastatic presentation was the strongest adverse prognostic factor, conferring significantly worse OS (*p* = 0.025) (Figure 8). Age-stratified Kaplan–Meier curves showed no significant survival differences between pediatric and adult patients (*p* = 0.25), despite a steeper visual decline in the adult cohort. No meaningful OS differences were observed by primary site (skeletal vs. extraskeletal).

Multimodal therapy was common across all age groups, with only modest age-related differences in treatment patterns. The most notable distinction was the use of neoadjuvant chemotherapy, which was significantly more frequent in younger patients (89.7% vs. 63.6%, *p* = 0.029). Adjuvant chemotherapy use did not differ significantly. The standard systemic regimen across all age groups was VDC/IE (vincristine, doxorubicin, cyclophosphamide alternating with ifosfamide/etoposide), consistent with contemporary Ewing sarcoma protocols. Definitive surgery was performed in most cases, with slightly higher utilization in younger adults, although this difference was not statistically significant. Radiation therapy use was comparable across age groups (56% in children vs. 48% in adults) and was primarily applied as an adjuvant treatment for close or positive margins or as definitive local control in unresectable disease.

Among ES patients, 24/76 (32%) experienced disease progression during therapy or follow-up. The median time to progression was 17.5 months (range 2.6–187.4 months). Most progression events occurred as distant metastases (*n* = 18), followed by isolated local recurrence (*n* = 5) and combined local–distant relapse (*n* = 1). Half of all progression events occurred in patients who initially presented with localized disease (12/24; 50%). Progression occurred in 11/41 (26.8%) children, 10/26 (38.5%) AYAs, and 3/9 (33.3%) older adults, with no statistically significant differences across these groups. In contrast, baseline metastatic presentation was strongly predictive of subsequent progression, with substantially higher rates in metastatic versus localized disease (12/18 [66.7%] vs. 12/55 [22%], *p* < 0.001).

## 4. Discussion

In this retrospective study, we explored age-related clinicopathological patterns in molecularly confirmed ES cases treated at two tertiary cancer centers in Kuwait. Understanding such patterns is clinically relevant for refining risk stratification and guiding age-tailored therapeutic strategies in a malignancy marked by rarity and biological complexity. To avoid diagnostic misclassification—a recurrent problem within the SRCS spectrum—only molecularly validated ES cases were included in the primary analyses. This approach is particularly important given the substantial morphologic overlap between ES, Ewing-like sarcomas, and non-sarcomatous small round blue cell neoplasms (such as carcinoma and lymphoma) [14]. As expected, *EWSR1::FLI1* was the dominant fusion type, followed by *EWSR1::ERG* and rare *FUS::ERG* events [15]. Beyond its diagnostic utility, *EWSR1::FLI1* may also establish targetable molecular dependencies, offering promising avenues for therapeutic innovation in this otherwise genomically sparse tumor [16,17,18].

Children (≤18 years) constituted the largest proportion of the ES cohort, consistent with the well-established age predilection of the disease. In this youngest subset, tumors predominantly arose in bone, mirroring classic pediatric presentations [19]. ES nevertheless remained distinctly uncommon at the extremes of pediatric age and was particularly rare in infancy (<12 months). This absence is epidemiologically meaningful, aligns with contemporary literature, and suggests that earlier registry-based reports of infantile ES lacking molecular confirmation may have included misclassified entities [20]. The predominance of soft-tissue primaries in such datasets further raises the possibility that at least a proportion may represent Ewing-like sarcomas (e.g., *BCOR*-altered tumors) or other pediatric non-sarcoma round-cell malignancies. Overall, pediatric ES cases are generally associated with more favorable baseline features, including lower rates of pelvic or axial primaries and metastatic disease, as reported in prior studies [21].

In contrast to younger children, adolescents and young adults frequently exhibit a higher burden of adverse prognostic factors, including pelvic or axial primary sites and a greater likelihood of metastatic presentation [20,22,23,24,25]. In our cohort, pelvic tumors represented the single most common primary site (*n* = 13) and clustered in the 2nd and 3rd decades of life, aligning with this age-related distribution. However, age-stratified survival curves did not demonstrate significant OS differences across age groups, likely reflecting limited event numbers and substantial censoring. Moreover, meaningful cross-study comparisons remain challenging because age cutoffs are inconsistently defined and often arbitrary, limiting the comparability of findings across the literature.

ES in older adults (>40 years) is rare [26] and diagnostically challenging, owing to both its atypical age range and its frequent presentation at nonclassical extraskeletal soft-tissue and visceral sites [27,28,29,30]. Imaging findings become less specific in these locations, reflecting variation in the anatomic context and the tissues involved [31]. Morphologic and immunophenotypic variability—such as nested, epithelioid, or rhabdoid patterns and aberrant cytokeratin, synaptophysin, or S100 expression—further complicates recognition [32,33,34]. Differential diagnosis may include carcinoma, melanoma, lymphoma, or other high-grade sarcomas. These pitfalls were evident in our cohort, as several extraskeletal small round cell neoplasms in older adults were ultimately excluded following pathological review, clinicopathological correlation, and molecular testing that excluded ES-specific fusions, thereby confirming alternative diagnoses. Such examples highlight the necessity of robust molecular testing in undifferentiated round-cell tumors arising in this age group, particularly those at atypical anatomical sites. Survival outcomes in patients >40 years are generally inferior to those of younger cohorts, reflecting a combination of unfavorable baseline features (advanced stage, pelvic involvement), potential intrinsic biological differences, and variation in treatment intensity or timing [35,36,37].

Visceral ES is exceptionally rare yet diagnostically important, having been reported across nearly all organ systems [38,39,40,41,42]. In our cohort, visceral ES accounted for 10.5% (8/76) of cases, involving the kidney (*n* = 5), small intestine, anorectum, and retroperitoneum. These tumors predominantly affected adults (mean age 34.8 years) and often mimicked site-specific malignancies. Diagnostic challenges included renal tumors with pseudopapillary or multicystic architecture and patchy cytokeratin expression resembling renal cell carcinoma, and a rectal tumor with marked cytoplasmic clearing mimicking metastatic clear cell carcinoma. A breast ES—though not strictly visceral—similarly resembled primary solid papillary carcinoma. All visceral tumors nonetheless harbored canonical FET::ETS rearrangements. Renal ES, the most common visceral subtype, exhibited large size, infiltrative growth, and potential venous extension into the renal vein or IVC, consistent with previous reports [42,43,44,45,46], with one case rapidly progressing to brain metastasis.

Metastatic presentation in ES remains a pivotal determinant of outcome. As reported in prior cohorts [47,48], approximately one-quarter of patients present with metastases, most commonly involving the lungs, bone, or marrow. Despite advances in therapy, metastatic and relapsed ES remain difficult to cure, with the notable exception of isolated pulmonary metastasis [49,50,51]. Although metastatic presentation has been reported to increase with age [23,24], this pattern was not evident in our cohort—likely reflecting limited statistical power within the older adult subgroup rather than a true absence of age-related risk. Nonetheless, overall metastatic rate (25%) aligned with published data, and metastatic disease remained the strongest adverse prognostic factor [48,52,53]. Age-stratified survival curves likewise showed no significant OS differences, with treatment patterns broadly similar except for more frequent neoadjuvant chemotherapy in younger patients.

Ewing-like SRCSs represent a rare but molecularly diverse group historically unified by round-cell morphology and prior inclusion within the ES spectrum [11]. In this study, they included *CIC::DUX4* [9,54], *BCOR*-altered [55], *EWSR1::ATF1/CREB1* [56], and *YWHAE::NUTM2B* sarcomas [57], as well as fusion-negative SRCSs, reflecting the expanding molecular landscape within this spectrum [58]. Collectively, these tumors showed a broader age range and lacked the adolescent peak typical of ES. While diagnostic familiarity and molecular immunohistochemistry are improving detection, definitive classification still hinges on molecular testing. *CIC*-rearranged sarcomas, the most frequent among *EWSR1*-negative SRCSs, show a strong soft-tissue predilection and significantly worse survival than ES [8]. Two infantile Ewing-like SRCSs were encountered, further exemplifying this heterogeneity—both extraskeletal involving the gluteal region: a 5-month-old with a *BCOR* exon 15 partial duplication who achieved complete remission and remains disease-free at 4.8 years of follow-up, and a neonate with a *YWHAE::NUTM2B* fusion who experienced rapid progression and died at 15 months. *BCOR*-ITD and *YWHAE*-rearranged tumors define an infantile SRCS subset with truncal/abdominopelvic predilection, aggressive behavior, and characteristic immunophenotypes (BCOR, cyclin D1, SATB2), overlapping morphologically with clear cell sarcoma of the kidney [57,59,60].

## 5. Conclusions

This study provides much-needed insight into age-linked anatomical and clinicopathological patterns within a fully molecularly validated national ES cohort. The modest sample size reflects the realities of institutional studies of an inherently rare tumor. Nevertheless, presenting a comprehensive, genetically confirmed series from the Middle East offers important regional context at a time when molecular diagnostics are being adopted with increasing consistency. Continued progress will require larger, collaborative, multinational registries to clarify age-specific biological features and refine treatment strategies across the ES age spectrum.

## Figures and Tables

**Table 1:** Characteristics of small round cell sarcoma cohort.

Characteristics	n (%)
**Gender**	
Female	43 (47.8%)
Male	47 (52.2%)
**Age**	
Mean (SD)	22.1 (14.2)
Range	0.0–69.7
**Age groups (binary)**	
≤18	45 (50.0%)
>18	45 (50.0%)
**Age groups (3-tier)**	
Children: 0 to 18	17 (18.9%)
AYAs: 19–39	30 (33.3%)
Older adults: ≥40	21 (23.3%)
**Tumor size (cm)**	
Mean (SD)	9.4 (5.1)
Range	1.4–29.0
**Size category**	
<8 cm	39 (44.8%)
≥8 cm	48 (55.2%)
**Anatomical region**	
Trunk	42 (46.7%)
Extremity	27 (30.0%)
Limb girdle	16 (17.8%)
Head and neck	5 (5.5%)
**Skeletal vs. extraskeletal**	
*Skeletal*	50 (55.6%)
Axial	29 (58.0%)
Appendicular	17 (34.0%)
Acral	3 (6.0%)
Craniofacial	1 (2.0%)
*Extraskeletal*	40 (44.4%)
Deep muscular compartment	14 (35.0%)
Subcutaneous/superficial compartment	11 (27.5%)
Visceral/retroperitoneal	11 (27.5%)
Head and neck	4 (10.0%)
**Pathological/molecular category**	
Ewing family (FET::ETS fusion)	76 (84.4%)
Ewing-like (alternative or no fusions)	14 (15.6%)
**Stage at diagnosis**	
Localized	62 (68.9%)
Metastatic	24 (26.7%)
Unknown	4 (4.4%)
**Total**	90

AYAs = Adolescents and young adults.

**Table 2:** Contrasting age groups in canonical Ewing sarcoma.

Variable	Children (*n* = 41)	AYAs (*n* = 26)	Older Adults (*n* = 9)	Total (*n* = 76)	*p* Value
**Gender**					0.410
Female	19 (46.3%)	15 (57.7%)	3 (33.3%)	37 (48.7%)	
Male	22 (53.7%)	11 (42.3%)	6 (66.7%)	39 (51.3%)	
**Tumor size**					0.219
<8 cm	21 (53.8%)	11 (44.0%)	2 (22.2%)	34 (46.6%)	
≥8 cm	18 (46.2%)	14 (56.0%)	7 (77.8%)	39 (53.4%)	
Unknown	2	1	0	3	
**Primary site**					0.016 *
Skeletal	30 (73.2%)	16 (61.5%)	2 (22.2%)	48 (63.2%)	
Extraskeletal	11 (26.8%)	10 (38.5%)	7 (77.8%)	28 (36.8%)	
**Baseline stage at diagnosis**					0.506
Localized	29 (72.5%)	20 (83.3%)	6 (66.7%)	55 (75.3%)	
Metastatic	11 (27.5%)	4 (16.7%)	3 (33.3%)	18 (24.7%)	
Unknown	1	2	0	3	
**Post-baseline progression**					0.603
Yes	11 (26.8%)	10 (38.5%)	3 (33.3%)	24 (31.6%)	
No	30 (73.2%)	16 (61.5%)	6 (66.7%)	52 (68.4%)	
**Neoadjuvant chemotherapy**					0.029 *
Yes	35 (89.7%)	15 (62.5%)	6 (66.7%)	56 (77.8%)	
No	4 (10.3%)	9 (37.5%)	3 (33.3%)	16 (22.2%)	
Unknown	2	2	0	4	
**Adjuvant chemotherapy**					0.318
Yes	29 (74.4%)	14 (58.3%)	5 (55.6%)	48 (66.7%)	
No	10 (25.6%)	10 (41.7%)	4 (44.4%)	24 (33.3%)	
Unknown	2	2	0	4	
**Definitive surgery**					0.294
Yes	25 (64.1%)	19 (82.6%)	6 (66.7%)	50 (70.4%)	
No	14 (35.9%)	4 (17.4%)	3 (33.3%)	21 (29.6%)	
Unknown	2	3	0	5	
**Radiation**					0.702
Yes	22/39 (56.4%)	10/22 (45.5%)	5/9 (55.6%)	37 (52.9%)	
No	17/39 (43.6%)	12/22 (54.5%)	4/9 (44.4%)	33 (47.1%)	
Unknown	2	4	0	6	
**Status last contact**					0.131
No evidence of disease	22/38 (57.9%)	8/24 (33.3%)	3/9 (33.3%)	33/71 (46.5%)	
Stable disease	9/38 (23.7%)	6/24 (25.0%)	4/9 (44.5%)	19/71 (26.8%)	
Progressive disease	2/38 (5.2%)	3/24 (12.5%)	2/9 (22.2%)	7/71 (9.8%)	
Dead	5/38 (13.2%)	7/24 (29.2%)	0/9 (0%)	12/71 (16.9%)	
Unknown	3	2	0	5	

Pearson’s Chi-squared test; unknowns excluded. AYAs = adolescents and young adults. * Indicates statistical significance.

## Data Availability

All data are contained within this article.

## Supplementary Materials

The following supporting information can be downloaded at: https://www.mdpi.com/article/10.3390/cancers18010133/s1, Table S1. FISH and RNA-based NGS results in canonical Ewing sarcoma and Ewing-like sarcomas.

## Abbreviations

The following abbreviations are used in this manuscript:

ES	Ewing sarcoma
SRCS	Small round cell sarcoma
