# Supplementary material for: Age-Related Clinicopathologic Patterns in Ewing Sarcoma (FET::ETS Family): A Comparative Analysis of Pediatric and Adult Patients
Source: Cancers (Basel). 2025 Dec 30;18(1):133. doi: 10.3390/cancers18010133 (PMC12784685; doi:10.3390/cancers18010133)
Supplement: Supplementary file 1 [file cancers-18-00133-s001.zip › cancers-4063891-supplementary.pdf]

Supplementary Table S1. FISH and RNA-based NGS results in canonical Ewing sarcoma and Ewing-like sarcomas.

Case ID	EWSR1 FISH performed	EWSR1 FISH result	RNA-NGS performed	Fusion detected by RNA-NGS	FISH/RNA-NGS discordance	Diagnostic category
SRCS-01	Yes	Pos	Yes	EWSR1::FLI1	—	ES
SRCS-02	Yes	Pos	Yes	EWSR1::FLI1	—	ES
SRCS-03	Yes	Pos	Failed	Failed	—	ES
SRCS-04	Yes	Pos	No	—	—	ES
SRCS-05	Yes	Pos	Failed	Failed	—	ES
SRCS-06	Yes	Pos	Yes	No fusion (scanty)	Yes	ES
SRCS-07	Yes	Pos	Yes	EWSR1::FLI1	—	ES
SRCS-08	Yes	Pos	Yes	EWSR1::FLI1	—	ES
SRCS-09	Yes	Pos	Yes	EWSR1::FLI1	—	ES
SRCS-10	Yes	Pos	Yes	EWSR1::FLI1	—	ES
SRCS-11	Yes	Pos	No	—	—	ES
SRCS-12	Yes	Pos	No	—	—	ES
SRCS-13	Yes	Pos	No	—	—	ES
SRCS-14	Yes	Pos	Yes	EWSR1::FLI1	—	ES
SRCS-15	Yes	Pos	No	—	—	ES
SRCS-16	Yes	Pos	No	—	—	ES
SRCS-17	Yes	Pos	No	—	—	ES
SRCS-18	Yes	Pos	Failed	Failed	—	ES
SRCS-19	Yes	Pos	Yes	No fusion (scanty)	Yes	ES
SRCS-20	Yes	Pos	Yes	No fusion (scanty)	Yes	ES
SRCS-21	Yes	Pos	Yes	EWSR1::FLI1	—	ES
SRCS-22	Yes	Pos	No	—	—	ES
SRCS-23	Yes	Pos	Yes	EWSR1::FLI1	—	ES
SRCS-24	Yes	Pos	Yes	EWSR1::ERG	—	ES
SRCS-25	Yes	Pos	Yes	EWSR1::FLI1	—	ES
SRCS-26	Yes	Pos	Yes	EWSR1::FLI1	—	ES
SRCS-27	Yes	Pos	Yes	No fusion (scanty)	Yes	ES
SRCS-28	Yes	Pos	Yes	EWSR1::FLI1	—	ES
SRCS-29	Yes	Pos	No	—	—	ES
SRCS-30	Yes	Pos	Yes	EWSR1::FLI1	—	ES
SRCS-31	Yes	Neg	Yes	EWSR1::FLI1	Yes	ES
SRCS-32	Yes	Pos	Failed	Failed	—	ES
SRCS-33	Yes	Pos	Yes	EWSR1::ERG	—	ES
SRCS-34	Yes	Pos	Yes	EWSR1::FLI1	—	ES
SRCS-35	No	Not done	Yes	EWSR1::FLI1	—	ES
SRCS-36	Yes	Pos	No	—	—	ES
SRCS-37	Yes	Pos	Yes	EWSR1::FLI1	—	ES
SRCS-38	Yes	Pos	Yes	EWSR1::FLI1	—	ES
SRCS-39	Yes	Pos	No	—	—	ES
SRCS-40	Yes	Pos	No	—	—	ES
SRCS-41	Yes	Pos	No	—	—	ES
SRCS-42	Yes	Pos	Yes	EWSR1::FLI1	—	ES
SRCS-43	Yes	Pos	Yes	EWSR1::FLI1	—	ES
SRCS-44	Yes	Pos	Yes	EWSR1::ERG	—	ES
SRCS-45	Yes	Pos	Yes	EWSR1::FLI1	—	ES
SRCS-46	Yes	Pos	Yes	EWSR1::FLI1	—	ES
SRCS-47	Yes	Pos	Yes	EWSR1::FLI1	—	ES
SRCS-48	Yes	Pos	No	—	—	ES
SRCS-49	Yes	Pos	Yes	EWSR1::FLI1	—	ES
SRCS-50	Yes	Pos	Yes	EWSR1::FLI1	—	ES
SRCS-51	Yes	Pos	No	—	—	ES

SRCS-52	Yes	Pos	Yes	EWSR1::FLI1	—	ES
SRCS-53	Yes	Pos	Yes	EWSR1::FLI1	—	ES
SRCS-54	Yes	Pos	No	—	—	ES
SRCS-55	Yes	Pos	Yes	EWSR1::FLI1	—	ES
SRCS-56	Yes	Pos	Failed	Failed	—	ES
SRCS-57	Yes	Pos	Yes	EWSR1::FLI1	—	ES
SRCS-58	Yes	Pos	Yes	No fusion (scanty)	Yes	ES
SRCS-59	Yes	Pos	Yes	EWSR1::FLI1	—	ES
SRCS-60	Yes	Pos	Yes	EWSR1::FLI1	—	ES
SRCS-61	Yes	Pos	Yes	EWSR1::FLI1	—	ES
SRCS-62	Yes	Pos	Failed	Failed	—	ES
SRCS-63	Yes	Pos	No	—	—	ES
SRCS-64	Yes	Pos	Failed	Failed	—	ES
SRCS-65	Yes	Neg	Yes	EWSR1::ERG	Yes	ES
SRCS-66	Yes	Neg	Yes	FUS::ERG	—	ES
SRCS-67	No	Not done	Yes	EWSR1::FLI1	—	ES
SRCS-68	Yes	Pos	No	—	—	ES
SRCS-69	Yes	Pos	Yes	EWSR1::FLI1	—	ES
SRCS-70	Yes	Pos	No	—	—	ES
SRCS-71	Yes	Pos	Yes	EWSR1::FLI1	—	ES
SRCS-72	Yes	Pos	Yes	EWSR1::FLI1	—	ES
SRCS-73	Yes	Pos	Failed	Failed	—	ES
SRCS-74	Yes	Pos	Yes	EWSR1::FLI1	—	ES
SRCS-75	Yes	Pos	Yes	EWSR1::FLI1	—	ES
SRCS-76	Yes	Pos	Yes	EWSR1::FLI1	—	ES
SRCS-77	Yes	Neg	Yes	BCOR alteration	—	ES-like
SRCS-78	Yes	Neg	Yes	YWHAE::NUTM2B	—	ES-like
SRCS-79	Yes	Neg	Yes	CIC::DUX4	—	ES-like
SRCS-80	Yes	Neg	Yes	CIC::DUX4	—	ES-like
SRCS-81	Yes	Neg	Yes	No fusion	—	ES-like
SRCS-82	Yes	Neg	Yes	No fusion	—	ES-like
SRCS-83	No	Not done	Yes	EWSR1::ATF1	—	ES-like
SRCS-84	Yes	Neg	Yes	No fusion	—	ES-like
SRCS-85	Yes	Neg	Yes	CIC::DUX4	—	ES-like
SRCS-86	Yes	Neg	Yes	CIC::DUX4	—	ES-like
SRCS-87	Yes	Neg	Yes	No fusion	—	ES-like
SRCS-88	Yes	Neg	Yes	No fusion	—	ES-like
SRCS-89	Yes	Neg	Yes	CIC::DUX4	—	ES-like
SRCS-90	Yes	Pos	Yes	EWSR1::CREB1	—	ES-like

Abbreviations:
FISH, fluorescence in situ hybridization; RNA-NGS, targeted RNA sequencing; ES, Ewing sarcoma; ES-like, Ewing sarcoma-like
